# Efficacy analysis of ligamentum flavum preservation technique under unilateral biportal endoscopic in the treatment of lumbar disc herniation

**DOI:** 10.3389/fsurg.2025.1627051

**Published:** 2025-10-20

**Authors:** Wen-Bo Wei, Sha-Jie Dang, Ling Wei, Da-Peng Duan

**Affiliations:** 1 Department of Orthopedics, Shaanxi Provincial People’s Hospital, Xi’an, Shaanxi, China; 2 Shaanxi Province Key Laboratory of Basic and Clinical Translation for Bone and Joint Diseases, Xi’an, Shaanxi, China; 3 State Key Laboratory for Manufacturing Systems Engineering, Xi’an Jiaotong University, Xi’an, Shaanxi, China; 4 Department of Anesthesiology, Shaanxi Provincial Cancer Hospital, Xi’an, Shaanxi, China; 5 Shaanxi Provincial Key Laboratory of Infection and Immune Diseases, Shaanxi Provincial People’s Hospital, Xi’an, China; 6 Department of Pain, The Third Affiliated Hospital of Xi'an Medical University, Xi’an, Shaanxi, China

**Keywords:** unilateral biportal endoscopic, ligamentum flavum, lumbar disc herniation, lumbar, clinical effects

## Abstract

**Background:**

Unilateral biportal endoscopic (UBE) surgery provides benefits like reduced invasiveness and swift recovery after surgery for individuals with lumbar disc herniation. A key factor in minimizing postoperative complications is the reduction of iatrogenic injury. This study retrospectively evaluates the effectiveness of the ligamentum flavum preservation technique during UBE for lumbar disc herniation and examines its technical application and clinical significance.

**Methods:**

From November 2023 to April 2024, 68 patients with lumbar disc herniation underwent unilateral biportal endoscopic (UBE) surgery via a single-side approach. Patients were allocated to either the conventional UBE group (Group T, *n* = 38) or the ligamentum flavum preservation group (Group P, *n* = 30) based on the surgical technique. Clinical outcomes were assessed using the Visual Analog Scale (VAS) for low back and leg pain, and the Oswestry Disability Index (ODI) at preoperative, postoperative 1, 3, 6 and 12 months. Operative time, length of hospitalization, postoperative drainage, the modified MacNab criteria, and complications were also recorded.

**Results:**

Postoperative VAS and ODI scores demonstrated significant improvement in both groups; however, no statistically significant differences were observed between them at 1 day, 1 month, 3 months, 6 months, or 12 months following surgery. At 6 months postoperatively, the incidence of epidural fibrosis was significantly lower in the ligamentum flavum preservation group (Group P) compared to the conventional UBE group (Group T). In Group T, one case of cerebrospinal fluid leakage and two cases of nerve root injury were reported; all complications were transient and resolved within three months. Overall, the complication rates during follow-up showed no significant intergroup differences (*P* > 0.05).

**Conclusions:**

The ligamentum flavum preservation technique applied during unilateral biportal endoscopic surgery enables effective removal of herniated disc material in cases of lumbar disc herniation, thereby relieving lower back pain and sciatica, enhancing lumbar function, reduce postoperative dural adhesions, and minimizing the risk of dural injury, cerebrospinal fluid leakage, epidural hematoma associated with ligamentum flavum resection.

## Introduction

Lumbar disc herniation (LDH), a common spinal disorder, presents with low back pain, sciatica, and neurological deficits, which exerts a detrimental effect on quality of life (1). Surgical intervention is considered appropriate when conservative modalities are insufficient to alleviate clinical symptoms (2, 3). Although traditional open discectomy remains an effective treatment modality, it presents notable limitations, such as extensive paraspinal muscle dissection, increased intraoperative blood loss, and extended postoperative recovery periods (4, 5). In contrast, minimally invasive techniques, such as unilateral biportal endoscopic (UBE) discectomy, have emerged as promising alternatives, offering reduced tissue trauma and faster recovery (6, 7).

The UBE technique synergizes the principles of conventional open surgery and endoscopic approaches through a dual-port system. One portal accommodates the endoscope and continuous saline irrigation, while the other facilitates the insertion of surgical instruments. This innovative setup enhances intraoperative maneuverability compared to single-portal endoscopy, providing surgeons with superior visualization and a wider working space for neural decompression. Moreover, UBE offers advantages in terms of rapid symptom relief and improved technical accessibility, potentially reducing the learning curve for spinal endoscopic procedures (8, 9).

Conventional unilateral biportal endoscopic (UBE) discectomy requires complete resection of the ligamentum flavum (LF), a procedure that carries intraoperative risks including dural injury, postoperative cerebrospinal fluid leakage, and the formation of epidural hematoma (10). Additionally, long-term complications, including epidural fibrosis and arachnoiditis, can result from LF removal (11). So LF is an important anatomical barrier for prevention of postoperative scar tissue (12) and mechanical stabilization of the lumbar segment (13). Preservation of the LF presents a strategic surgical alternative, especially beneficial for mitigating challenges in revision surgeries by minimizing scar tissue formation (14).

This study introduces an innovative ligamentum flavum preservation technique designed to balance neural decompression with anatomical protection. Through targeted detachment of the lateral LF margin combined with intraspinal nerve exposure, the technique achieves two main objectives: (1) adequate decompression of neural structures, and (2) preservation of LF integrity to maintain its biomechanical barrier function. The present article evaluates the clinical outcomes of this refined approach.

## Methods

### Study design

A retrospective analysis was performed on the medical records of 68 patients who underwent surgical treatment for lumbar disc herniation at the Department of Orthopedics, Shaanxi Provincial People's Hospital, between November 2023 and April 2024. The study received approval from the Clinical Research Ethics Committee of the same institution (Approval No. 2023-019) and complied with Good Clinical Practice (GCP) guidelines and the ethical principles of the Declaration of Helsinki. Of the enrolled patients, 38 underwent conventional ligamentum flavum resection (Group T), while 30 received the ligamentum flavum preservation technique (Group P).

### Patients

This study included patients with lumbar disc herniation (LDH) who underwent either complete excision of the ligamentum flavum or the ligamentum flavum preservation technique. Prior to surgery, the advantages, disadvantages, and potential complications of both approaches were fully explained to the patients, who then selected their preferred approach. All procedures were performed by two spine surgeons, each with over 10 years of experience. The inclusion criteria were: (1) single-level disc herniation at L4/L5 or L5/S1; (2) lack of response to conservative treatment for at least three months; (3) presence of low back pain and/or sciatica; and (4) radiological confirmation via MRI and CT imaging. Exclusion criteria comprised: (1) multi-level disc herniation, cauda equina syndrome, lumbar spinal stenosis, spinal metastatic disease, or lumbar spondylolisthesis; (2) psychiatric disorders or uncorrectable bleeding disorders; (3) patients lost to follow-up; and (4) history of prior lumbar surgery.

## Procedures

### Surgical technique (traditional UBE)

Under C-arm fluoroscopic guidance, the patient was positioned prone on U-shaped bolsters placed beneath the chest and iliac crests to unload the abdomen. The target intervertebral level (left side) was localized fluoroscopically, after which two 1.0 cm skin incisions were made 3 cm apart, centered over the disc space. Guide rods were introduced through these incisions and advanced to the junction of the superior lamina and inferior articular process, with final placement confirmed by fluoroscopy.

**Figure 1:**
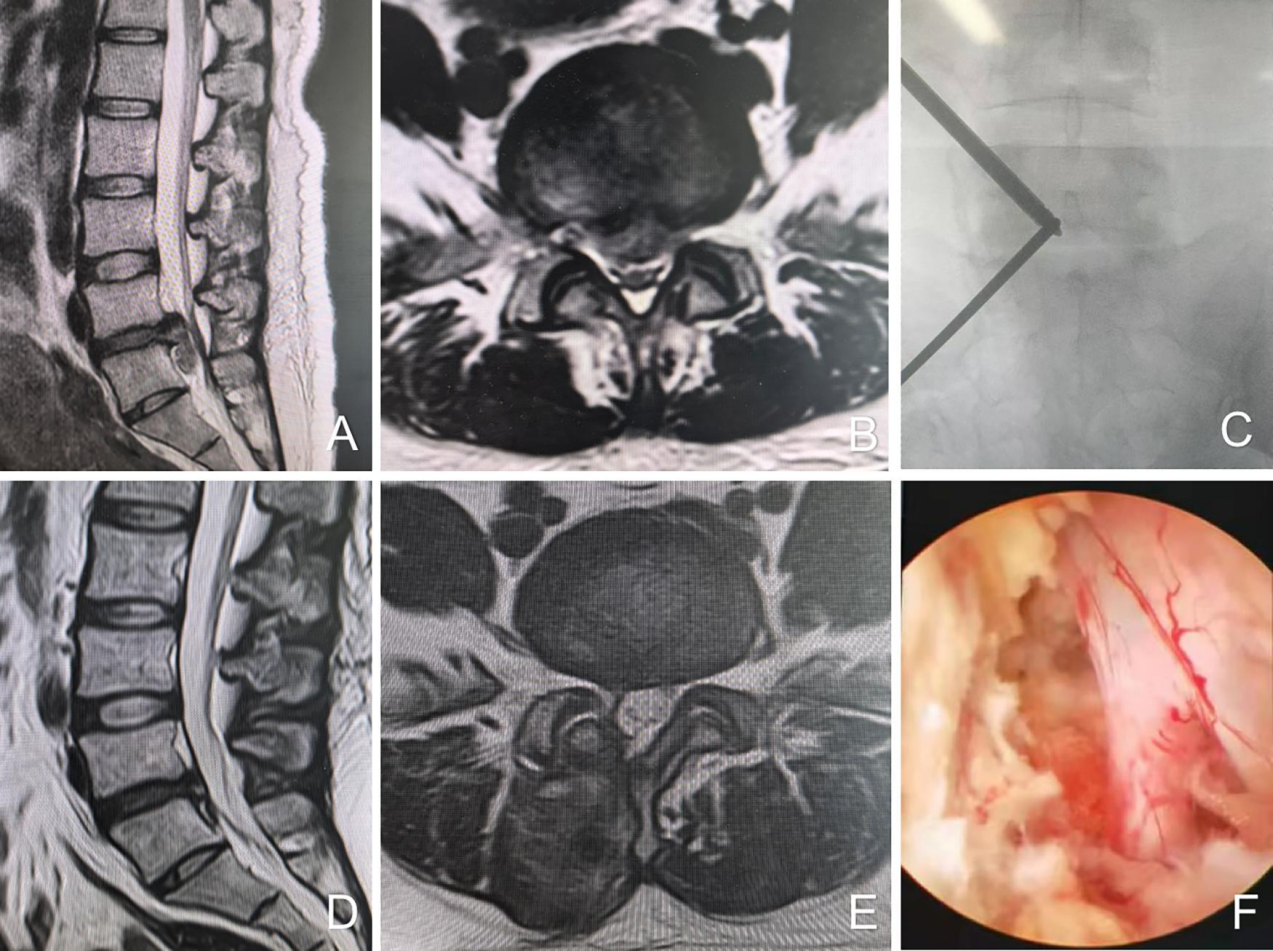
Female, 39 years old, L5/S1 lumbar disc herniation. **(A)** Preoperative sagittal MR image showed L5/S1 lumbar disc herniation; **(B)** preoperative axial MR image showed herniated lumbar disc compressed nerve root and dural sac; **(C)** intraoperative fluoroscopic confirmation of metal rods; **(D)** postoperative sagittal MR image shows complete decompression of the spinal canal, but with intraspinal adhesions; **(E)** postoperative axial MR image showed the complete removal of herniated disc and bony fragment; **(F)** intraoperative image after complete neural decompression.

A T-shaped dilator is employed for blunt dissection of the soft tissue. The cranial portal (observation channel) is utilized for the insertion of the endoscope (Stryker, Kalamazoo, MI, USA) to provide visualization, while the caudal portal (working channel) serves for the introduction of surgical instruments and radiofrequency (RF) ablation equipment (BONSS, Jiangsu, China). RF ablation and pituitary forceps are used to clear soft tissue within the visual field, thereby exposing the superior and inferior laminae, articular processes, and the base of the spinous process. Partial laminotomy is performed using a high-speed grinding drill (Xishan, Tianjin, China) and Kerrison punches to expose the insertion site of the ligamentum flavum. The ligamentum flavum is resected using Kerrison punches to reveal the underlying dura mater and nerve roots. With gentle medial retraction of the traversing nerve root, discectomy is performed using pituitary forceps. A neural probing hook is employed to confirm the absence of residual disc fragments. Following meticulous hemostasis, a drainage tube is placed, and the surgical incisions are closed with sutures (Figure 1).

### Ligamentum flavum preservation technique

**Figure 2:**
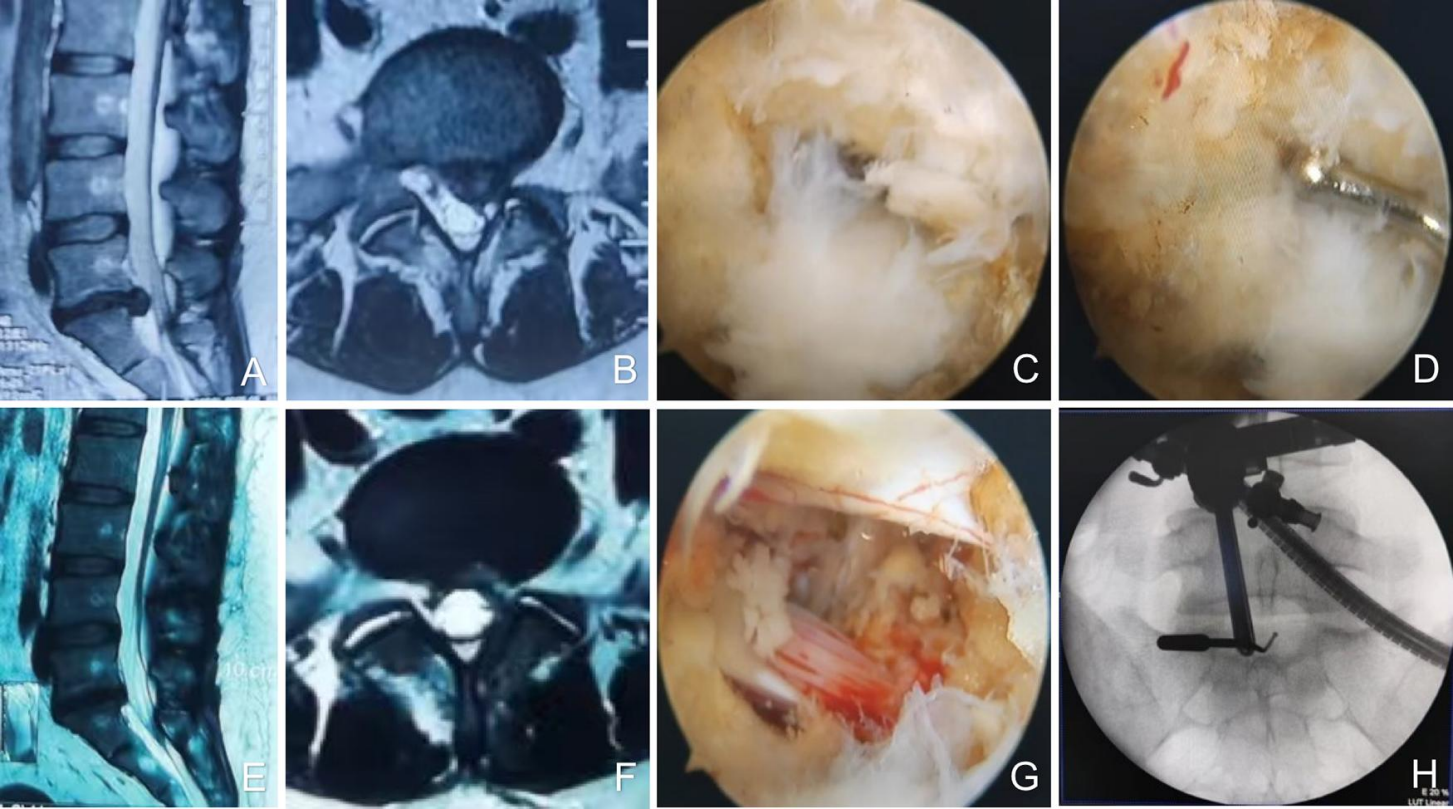
Female, 36 years old, L5/S1 lumbar disc herniation. **(A)** Preoperative sagittal MR image showed L5/S1 lumbar disc herniation; **(B)** preoperative axial MR image showed herniated lumbar disc compressed nerve root and dural sac; **(C)** intraoperative ligamentum flavum splitting line; **(D)** the fissure in the ligamentum flavum is only 2 mm; **(E)** postoperative sagittal MR image revealed the complete decompression of the spinal canal; **(F)** postoperative axial MR image showed the complete removal of herniated disc and bony fragment; **(G)** intraoperative image after complete neural decompression; **(H)** intraoperative fluoroscopy shows decompression crossing the midline.

In the ligamentum flavum preservation technique, only the medial margin of the inferior articular process and a limited portion of the inferior border of the superior lamina are exposed to establish the extra-spinal canal working space. Subsequently, bone resection is carried out medial to the superior articular process, at the level corresponding to the medial border of the pedicle. Upon exposure of the shoulder of the traversing nerve root, it is carefully retracted toward the midline to facilitate visualization and removal of the herniated disc material. Thereafter, thermal coagulation is applied to the annulus fibrosus to minimize the risk of recurrence. The adequacy of nerve root decompression and the preservation status of the ligamentum flavum are then evaluated endoscopically. The procedure is completed after conforming the complete decompression and freely movement of nerve root. Following confirmation, a drainage tube is placed. Finally, the working cannula and endoscope are withdrawn, and the surgical incision is meticulously closed with sutures (Figure 2).

### Postoperative treatment

All patients were instructed to wear a lumbar brace during ambulation following surgery. During the first postoperative month, they were advised to avoid weight-bearing activities and to perform moderate exercises aimed at strengthening the lumbar paraspinal muscles.

### Outcomes

All surgery-related complications were systematically recorded. Outcome assessment was completed by research members who were trained before the study and not involved in the clinical care of the patients. Pain intensity in the lower back and legs, as well as lumbar functional status, was assessed at 1 day, 3 months, 6 months, and 1 year postoperatively using the Visual Analog Scale (VAS) and the Oswestry Disability Index (ODI), respectively. Nerve root function was evaluated at 1 year after surgery using the MacNab criteria. Preoperative and 6-month postoperative lumbar spine magnetic resonance imaging (MRI) was performed using a 1.5-T scanner, slice thickness was 4 mm. The extent of epidural fibrosis in each quadrant was quantitatively evaluated by two independent radiologists in a double-blinded manner. five contiguous axial slices (centered on the intervertebral disc) were subdivided into four quadrants, defined by perpendicular lines through the center of the thecal sac. Epidural scarring was classified as Grade 1 (<25% involvement) or Grade 2 (>25% involvement).

### Statistical analysis

Statistical analyses were conducted using SPSS version 24.0 for Windows (IBM Corp., Armonk, NY, USA). Continuous variables were presented as mean ± standard deviation (SD) and compared using the independent-samples *t*-test. Repeated measures analysis of variance (ANOVA) with Bonferroni correction was applied to evaluate differences in VAS and ODI scores over time. Categorical variables were expressed as counts (*N*) and percentages (%) and analyzed using the chi-squared (*χ*^2^) test. A *p*-value of <0.05 was considered statistically significant.

## Results

### General information

No statistically significant differences were observed between the two groups with respect to sex, age, body mass index (BMI), or classification of disc herniation (*P* > 0.05). Additionally, there were no significant intergroup differences in operative time (56.66 ± 10.972) (53.17 ± 10.212), frequency of intraoperative fluoroscopy (3.32 ± 0.662) (3.53 ± 0.681), length of hospital stay (7.53 ± 2.102) (7.50 ± 2.556), or incidence of complications (10.5%) (10%).

None of the patients in either group experienced severe complications such as spinal injury or paraplegia. In Group P, one patient experienced nerve root injury, and three patients had cerebrospinal fluid (CSF) leakage. In Group T, one patient experienced CSF leakage, and two patients had nerve root injury. All complications were reversible and resolved within 3 months. The complication rates during follow-up did not differ significantly between the two groups (*P* = 0.944) (Table 1).

**Table 1:** Comparison of general data between Group T and Group P.

Characteristic		Group T (*n* = 38)	Group P (*n* = 30)	*t*/(*x*^2^)	*P*
Male/female		17/21	14/16	0.025	0.874
Age (years)		57.61 ± 14.269	58.30 ± 14.798	−0.196	0.845
BMI (kg/m^2^)		26.15 ± 4.13	27.06 ± 3.49	−0.039	0.827
Disc herniation classification	Protrusion	22	18	0.031	0.861
	Extrusion	16	12		
Hospitalization time (days)		7.53 ± 2.102	7.50 ± 2.556	0.047	0.963
Operation time (min)		56.66 ± 10.972	53.17 ± 10.212	1.343	0.184
Complication		4 (10.5%)	3 (10%)	(0.005)	0.944
Fluoroscopy frequency		3.32 ± 0.662	3.53 ± 0.681	−1.328	0.189

Numeric data were expressed as Mean ± SD and analyzed by Independent-Samples *T*-test. Categorical data were expressed by the number of patients (%) and were analyzed with the *χ*^2^ test. Group T: the traditional UBE group, Group P: the ligamentum flavum preservation group.

BMI, body mass index; UBE, unilateral biportal endoscopic.

### Comparison of VAS

No significant difference was observed in preoperative VAS scores for low back pain and sciatica between the two groups. In both groups, VAS scores for low back pain and sciatica at all postoperative follow-up time points showed a significant reduction compared to preoperative values (*P* < 0.05). However, there were no statistically significant differences in VAS scores between the groups at 1, 3, 6, and 12 months postoperatively (*P* > 0.05) (Table 2).

**Table 2:** Comparison of VAS between Group T and Group P at different time.

Group	VAS score	Pre-operation	Post-operation				
			1 day	1 month	3 months	6 months	12 months
Group T (*n* = 38)	Low back pain	7.84 ± 0.13	2.13 ± 0.13*	1.84 ± 0.12*	1.55 ± 0.10*	1.21 ± 0.11*	0.71 ± 0.11*
	Sciatica	7.79 ± 0.17	2.18 ± 0.12*	1.66 ± 0.14*	1.45 ± 0.11*	1.21 ± 0.11*	0.63 ± 0.11*
Group P (*n* = 30)	Low back pain	7.73 ± 0.15	2.13 ± 0.15*	1.90 ± 0.13*	1.67 ± 0.16*	1.13 ± 0.13*	0.60 ± 0.12*
	Sciatica	7.50 ± 0.19	2.20 ± 0.14*	1.77 ± 0.16*	1.67 ± 0.13*	1.13 ± 0.13*	0.57 ± 0.12*
Time F, *P*	Low back pain	883.912, <0.001					
	Sciatica	708.943, <0.001					
Group F, *P*	Low back pain	0.778, 0.310					
	Sciatica	0.810, 0.543					
Time* Group F, *P*	Low back pain	0.338, 0.890					
	Sciatica	0.638, 0.671					

* *P* < 0.05.

Data are presented as mean ± SD. The groups were compared by repeated measures analysis of variance (ANOVA). Bonferroni correction was used to correct multiple comparisons. Group T: the traditional UBE group, Group P: the ligamentum flavum preservation group. vs. pre-operation in the same group.

VAS, visual analog scale; UBE, unilateral biportal endoscopic.

### Comparison of ODI

There was no significant difference observed in ODI score at the pre-operation between the two groups. There was no statistical difference in ODI between the two groups at 1, 3, 6 and 12 months of the post-operation (*P* > 0.05) (Table 3).

**Table 3:** Comparison of ODI between Group T and Group P at different time.

Group	Pre-operation	Post-operation				
		1 day	1 month	3 months	6 months	12 months
Group T (*n* = 38)	82.71 ± 1.17	25.53 ± 0.97*	22.00 ± 0.87*	19.90 ± 0.94*	17.47 ± 0.78*	10.50 ± 0.46*
Group P (*n* = 30)	80.10 ± 1.32	24.10 ± 1.10*	21.43 ± 0.98*	19.30 ± 1.06*	15.47 ± 0.88*	10.40 ± 0.69*
Time F, *P*	1,495.735, <0.001					
Group F, *P*	0.488, 0.785					
Time* Group F, *P*	4.396, 0.112					

* *P* < 0.05.

Data are presented as mean ± SD. The groups were compared by repeated measures analysis of variance (ANOVA). Bonferroni correction was used to correct multiple comparisons. Group T: the traditional UBE group, Group P: the ligamentum flavum preservation group. vs. pre-operation in the same group.

ODI, oswestry disability index; UBE, unilateral biportal endoscopic.

### Imaging outcomes

At 6 months after surgery, there was significant difference of Epidural scarring detected in MRI between two groups. The grade of fibrosis in Group P was significantly lower than that in Group T (*P* < 0.001) (Table 4).

**Table 4:** Distribution of fibrosis at 6 months post-operative.

Grade of fibrosis	Group T (*n* = 38)	Group P (*n* = 30)	*x* ^2^	*P*
Grade 1	10	25	21.82	<0.001
Grade 2	28	5		

Statistical significance was set at *P* < 0.05. Epidural scarring was graded as Grade 1 (less than 25%) and Grade 2 (greater than 25%).

### MacNab criteria

According to the modified MacNab criteria, the (Excellent/Good/Fair/Poor) for each group at 12 months were 13,20,3,2 and 15,13,1,1, respectively. The rates of excellent and good outcomes at 12 months were 86.8% in the UBE-T group and 93.3% in the UBE-P group, with no significant difference between the two groups (*P* = 0.5625) (Table 5).

**Table 5:** Comparison of follow-up outcomes in Group T and Group P.

Modified MacNab score	Group T (*n* = 38)	Group P (*n* = 30)	*x* ^2^	*P*
Excellent	13	15	2.048	0.5625
Good	20	13		
Medium	3	1		
Poor	2	1		

## Discussion

Lumbar disc herniation (LDH), a primary cause of low back pain and sciatica, significantly impairs daily functioning and quality of life, thereby necessitating timely intervention to alleviate pain and restore functional capacity (1, 3). While minimally invasive surgeries are increasingly preferred due to their rapid recovery benefits (6), this study introduces a UBE-guided ligamentum flavum preservation technique. This technique only exposes the inner edge of the lower articular process. By grinding the inner edge of the inferior articular process, the outer margin of the ligamentum flavum is exposed, allowing for lateral separation of the ligamentum flavum. Arthroscopy is performed to enter the spinal canal for discectomy. Preliminary results suggest that this technique reduces both intraoperative complications (e.g., dural tears, epidural hematoma) and long-term complications (e.g., fibrosis, arachnoiditis) compared to standard UBE procedures, while maintaining the LF's biomechanical function as a barrier to postoperative adhesion formation.

A 15-year study involving 500 patients who underwent lumbar discectomy reported that 87.3% of patients had good to very good outcomes at one-year follow-up. However, outcomes deteriorated over time, with 63.7% remaining satisfactory after an average follow-up of 14.7 years (6). It is established that the success rate of discectomy declines with prolonged follow-up. Several studies on microendoscopic lumbar discectomy have indicated that resection of the ligamentum flavum can lead to spinal dural adhesions and arachnoiditis, both of which are major contributors to failed back surgery syndrome (10, 15). These spinal dural adhesions may be responsible for long-term functional deterioration and recurrent radicular symptoms in patients who have undergone discectomy.

The ligamentum flavum (LF) serves as a crucial anatomical barrier, preventing postoperative scar tissue formation and providing mechanical stabilization to the lumbar segment (16). The ligamentum flavum (LF) is a well-characterized elastic structure, consisting of approximately 80% elastin and 20% collagen fibers (17). The ligamentum flavum exhibits unique elastic properties that are essential for maintaining spinal stability and enabling the spine to return to a neutral position after flexion (18). Moreover, its elastic fibers help resist excessive flexion during extension, thereby preventing a reduction in spinal canal volume. Preservation of the ligamentum flavum not only reduces postoperative scar tissue formation but also assists surgeons in identifying anatomical landmarks during revision procedures (19, 20).

During LF removal, there is a risk of dural tear, which may lead to cerebrospinal fluid (CSF) leakage (21, 22). Patients with dural tears incur 120% higher medical costs, experience a 200% increase in hospital stay duration, and have twice the likelihood of readmission (23). Therefore, the surgeon's anatomical expertise and meticulous technique are critical in minimizing the risk of CSF leakage (24). In the traditional group, superficial layers of the ligamentum flavum (LF) are initially removed using nucleus pulposus forceps, followed by the application of a grinding drill to excise the edges of the lamina and articular process, thereby exposing the insertion point of the LF. A nerve dissector is then used to separate the LF margins, after which the deep layer is completely removed using Kerrison punches or nucleus pulposus forceps. Thoroughly expose the dura mater and nerve roots. In the preservation group, only the lateral edge of the LF is exposed, and a nerve dissector is employed to bluntly separate and open the fissure of the LF, exposing the outer edge of the nerve root. The subsequent removal of lateral LF tissue is determined intraoperatively based on the adequacy of neural structure visualization. In our study, nerve root injury was primarily observed during the early phase of surgical implementation. This complication ceased as the surgical team's experience increased over time.

Removal of the ligamentum flavum (LF) can lead to intraspinal adhesions and epidural scarring, both of which are recognized risk factors contributing to postoperative residual symptoms (25). Excessive fibrosis at the postoperative epidural bed may lead to neural irritation or stretching, and in some cases, mass effect, resulting in radicular symptoms. Although open surgery allows for full exposure of spinal canal structures and is effective in treating lower back and leg pain, it carries a high risk of complications such as intraspinal adhesions and epidural scarring. Postoperative epidural scarring disrupts the normal epidural architecture, particularly replacing the protective adipose tissue that aids in the physiological gliding of the dura mater and nerve roots. This creates mechanical tethering effects, restricting the mobility of neural structures, and leading to two pathological consequences: dynamic compression due to impaired nerve root excursion during spinal motion, and static compression from direct scarring effects. Both mechanisms compromise neural microcirculation and trigger chronic inflammatory responses, ultimately manifesting as recurrent radicular pain syndromes (12). Therefore, avoiding or minimizing intraspinal adhesions and epidural scarring is crucial for improving postoperative clinical outcomes. Preserving the ligamentum flavum during discectomy can help minimize intraspinal adhesions and epidural scarring (12, 26). Although complete prevention of epidural scarring is not possible, efforts to minimize its occurrence during discectomy have underscored the clinical importance of preserving the ligamentum flavum and support the adoption of endoscopic spine surgery. These techniques reduce epidural scarring by limiting the extent of discectomy and ligamentum flavum resection (26–28). Previous studies have shown that preserving the ligamentum flavum can reduce the occurrence of postoperative adhesions (6). In our study, the ligamentum flavum preservation group aimed to reduce intraspinal adhesion formation by preserving the ligamentum flavum. Postoperative MRI conducted three months after surgery revealed intact preserved ligamentum flavum and no significant intraspinal adhesions in the preservation group.

The ligamentum flavum preservation technique does not compromise the completeness of lumbar spinal canal, as it preserves the ligamentum flavum and involves relatively limited laminectomy (29). Lumbar disc herniation (LDH) predominantly occurs at the L4/5 and L5/S1 levels, where larger interlaminar spaces offer an anatomical advantage for surgical access to the spinal canal with minimal resection of bony structures, thus reducing the risk of postoperative lumbar instability (30, 31). Based on this rationale, our study specifically focused on the L4/5 and L5/S1 levels as the target surgical segments. After one year of follow-up, we found that the ligamentum flavum preservation technique during unilateral biportal endoscopic lumbar discectomy effectively reduced lower back and leg pain and improved lumbar function, with outcomes comparable to those of the conventional approach. Moreover, 94.28% of patients achieved “excellent” or “good” MacNab scores, which were superior to those observed following conventional surgery. However, the occurrence of incomplete removal of prominent disc material in Group P highlighted that blind spots and decreased visualization may be a limitation of the ligamentum flavum preservation technique. The blind spot created by the ligamentum flavum preservation technique may have led to missed disc fragments. Although transient weakness occurred in 2 patients in the split-group, it is possible that nerve root injury occurred during the Group P. Thus, preoperative planning and careful surgical technique are critical to avoid such complications.

This study has several limitations that warrant consideration. Firstly, its retrospective design introduces potential biases, including those related to patient selection and incomplete clinical data. Secondly, the investigation was confined to a single center with a relatively small sample size, which may limit the generalizability of the results. Future large-scale, multicenter prospective studies are required to further confirm and expand upon these findings.

## Conclusion

The ligamentum flavum preservation technique under unilateral biportal endoscopic (UBE) enables effective removal of herniated disc material, alleviation of lower back pain and sciatica, reduce postoperative dural adhesions, while reducing the risks of dural tears and cerebrospinal fluid leakage associated with ligamentum flavum resection.

## Data Availability

The original contributions presented in the study are included in the article/Supplementary Material, further inquiries can be directed to the corresponding author.
